# Authors' reply

**Published:** 2010

**Authors:** Pramod Bhende, Lingam Gopal, Tarun Sharma, Aditya Verma, Rupak Kanti Biswas

**Affiliations:** Sri Bhagwan Mahaveer Vitreoretinal Services, Medical and Vision Research Foundations, Sankara Nethralaya, Chennai, India

Dear Editor,

The authors wish to thank Shah *et al*.[1] for their keen interest in our article,[2] which extends the discussion on the particular matter. We appreciate the comments and suggestions written by them regarding the surgical management of Stage 4 and 5 retinopathy of prematurity (ROP). We would like to discuss the issues raised regarding the subject.

1. We completely agree that the term'pars plicata vitrectomy' is more aptly applied to these small eyes, in which the pars plana is incompletely developed.[3]
2. Stage 4A only indicates attached macula and may have a localized traction along the ridge in the periphery, or may have extensive proliferation with tractional component all along the ridge. Stage 4A eyes require surgical intervention only when the retinal detachment progresses, or when there is no desired response despite adequate laser ablation.[4] All our study eyes with Stage 4A ROP had progressive disease.
3. Iatrogenic break formation during the vitreoretinal surgery in ROP generally carries a poor prognosis in terms of anatomical success. Of the three eyes with iatrogenic breaks in our series, two had a break in previously lasered retina. In both these eyes, additional retinopexy was performed during the surgery. Third eye was noted to have a break nasal to the disc and reported with retinal detachment at two months follow-up. Subsequently it underwent re-surgery with successful outcome.
4. Authors fully agree that the 23- or 25-gauge systems have made life easier with much improved maneuverability of instruments in these small eyes. Suturing of sclerotomies can be left to the surgeon's discretion.[5] The majority of our cases reported in this series were operated before 23G or 25G systems were established in clinical practice. Presently, nearly all the lens-sparing vitrectomies for Stage 4 and some of the Stage 5 ROP in our institute are being carried out with the smaller gauge instruments. We routinely suture the 23G sclerotomies including overlying conjunctiva. However, we leave the 25G sclerotomies unsutured, and have never had a postoperative hypotony or sclerotomy-related complications in these eyes.
5. Triamcinolone was used at the end of the surgery by Lakhanpal *et al*. for vascularly active Stage 5 ROP eyes for limiting the postoperative inflammation and re-proliferation, and not for the better visualization of vitreous during the surgery.[6] In Stage 5 ROP, generally there is extensive proliferation and vitreous membranes without much gel, which does not need additional dye for visualization. We completely agree with[1] and routinely use triamcinolone for better visualization of the vitreous during the surgery in Stage 4 ROP eyes.
